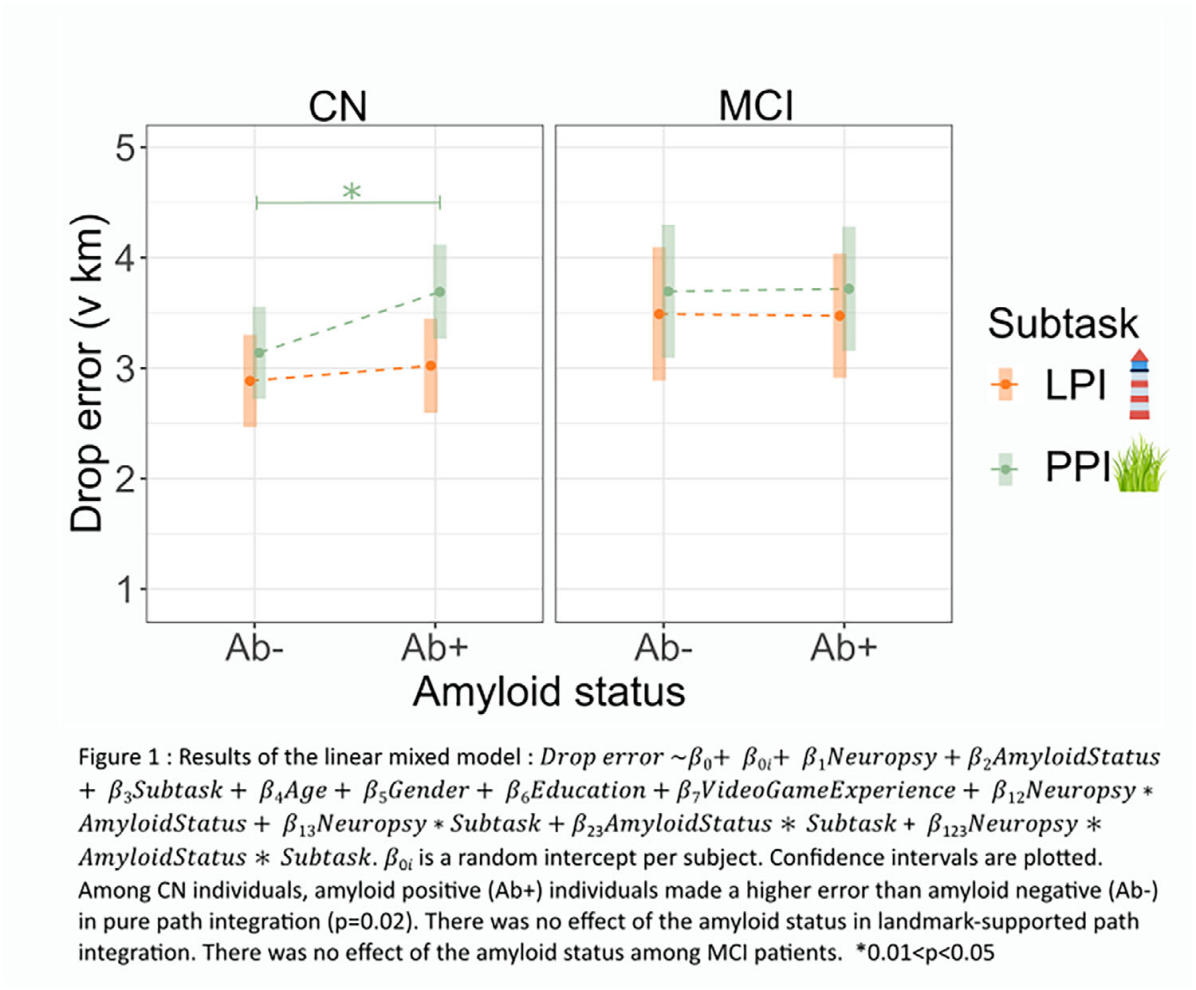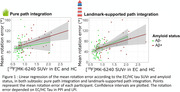# Path integration deficits are related to Alzheimer’s disease pathology

**DOI:** 10.1002/alz.086877

**Published:** 2025-01-03

**Authors:** Lise Colmant, Emilien Boyer, Thomas Gérard, Lara Huyghe, Lisa Quenon, Lukas Kunz, Nikolai Axmacher, Philippe Lefèvre, Bernard J Hanseeuw

**Affiliations:** ^1^ Institute of Neuroscience, UCLouvain, Brussels Belgium; ^2^ Institute of Neuroscience ‐ UCLouvain, Brussels Belgium; ^3^ University Hospital Bonn, Bonn Germany; ^4^ Institute of Cognitive Neuroscience, Ruhr University Bochum, Bochum Germany; ^5^ UCLouvain, Louvain‐La‐Neuve Belgium

## Abstract

**Background:**

The medial temporal lobe (MTL) is the first cortical region affected by tauopathy in Alzheimer’s disease (AD) and is implicated in spatial orientation. In early AD stages, navigation deficits, including path integration deficits, could be present, even before memory deficits. We investigated whether these deficits were related to AD pathology (amyloidosis and/or tauopathy) using a path integration task, the “Apple Game”.

**Method:**

During the task, participants navigated through a virtual arena and were asked to return to a starting location after visiting one intermediate location. Two subtasks were tested: with a proximal cue (landmark‐supported path integration, LPI), and without (pure path integration, PPI). We recruited 90 participants (mean age: 69): 66 clinically normal (CN) and 24 patients with mild cognitive impairment (MCI). We classified them according to amyloid status: Aβ+ (n = 34) or Aβ‐ (n = 56) based on amyloid‐PET or CSF measurements. All participants underwent [^18^F]MK‐6240 Tau‐PET to disclose tau pathology in the MTL (entorhinal cortex and hippocampus). Task performances were analyzed in relation to amyloid status and MTL tauopathy using linear‐mixed‐effect models. First, we evaluated the error according to amyloid status, neuropsychological status, subtask, and their interactions. Then, we evaluated the error and its components (rotation and distance errors) according to MTL tauopathy, and amyloid status for each subtask. Education, gender, and video game experiences were adjusted for.

**Result:**

Preclinical AD individuals (CN, Aβ+) had a deficit in pure path integration (p = 0.02) compared to CN Aβ‐ individuals, but not in landmark‐supported path integration (Fig. 1). The amyloid status did not influence performances among MCI patients. Rotation errors depended on MTL tauopathy in PPI (p = 0.02) and LPI (p = 0.0004) (Fig. 2), whereas distance errors depended on age in PPI (p = 0.004), and on the age (p = 0.015) and MTL tauopathy (p = 0.02) in LPI.

**Conclusion:**

We detected a specific deficit in pure path integration in a virtual environment, in preclinical AD, suggesting a deficit to integrate self‐motion cues. Furthermore, we decomposed the path integration performances into rotation and distance errors, and found that rotation errors were associated with MTL tauopathy, possibly reflecting impairment of grid cells; whereas distance errors mainly increased with older ages.